# Bridging the writing gap in studying language related disorders: the process and the product

**DOI:** 10.3389/fpsyg.2023.1292602

**Published:** 2023-12-05

**Authors:** Åsa Wengelin, Ingrid Henriksson, Luuk Van Waes

**Affiliations:** ^1^Department of Swedish, Multilingualism, Language Technology, University of Gothenburg, Gothenburg, Sweden; ^2^Department of Health and Rehabilitation, University of Gothenburg, Gothenburg, Sweden; ^3^Faculty of Business Economics, Department of Management, University of Antwerp, Antwerp, Belgium

**Keywords:** writing difficulties, writing processes, language disorder, cognitive disorders, dyslexia, keystroke logging, hearing impairment, handwriting recording

Proficiency in writing is essential for effective functioning in today's digitalized world. As pointed out by, for instance, Brandt (2014) and Rønneberg (2018), the world is full of text, and as society becomes increasingly digitalized the demand for written communication grows substantially. In the Western world, we find ourselves writing and possibly debating students' writing skills more frequently than ever before. Writing not only constitutes a vital competency across various academic subjects but also serves as a pivotal tool in professional settings and as a prerequisite for active engagement within democratic societies. These evolving dynamics pose formidable challenges for individuals grappling with language-related difficulties, ranging from developmental language disorders and dyslexia to aphasia and dementia. In fact, many individuals contending with language difficulties express writing as their most significant area of struggle (Connelly et al., 2006). This also extends to individuals with other disorders that might impede the acquisition of written language, such as hearing impairments (Breland et al., 2022).

For those with developmental challenges, like dyslexia or developmental language disorder (DLD), it is common for writing difficulties to persist even after overcoming reading obstacles (Berninger and Amtmann, 2003). Likewise, individuals recovering from post-stroke aphasia often find that writing difficulties linger long after regaining other capabilities and completing rehabilitation. The loss of writing proficiency is deeply felt, greatly impacting the quality of life and sense of independence for affected individuals (Parr, 2007; Knollman-Porter et al., 2015; Kjellén et al., 2017; Thiel and Conroy, 2022).

Despite successful research in identifying potential causes and solutions for reading challenges, writing difficulties surprisingly remain largely un(der)explored (Connelly et al., 2006). Back in 2008, Berninger et al., for instance, published a paper titled “*Writing problems in developmental dyslexia: under-recognized and under-treated*,” shedding light on the often disregarded and inadequately addressed nature of writing issues in those with developmental dyslexia. They emphasized not only the under-recognition of writing difficulties associated with dyslexia but also the necessity for comprehensive assessments and targeted interventions. They argued that heightened awareness among educators, parents, and the broader community regarding the impact of writing challenges was crucial for effectively addressing writing impediments in individuals with dyslexia and for developing efficient support and intervention strategies. Nevertheless, subsequent to the publication by Berninger et al. (2008), the need for deeper understanding has intensified, rather than diminished. Although the escalating demand for written communication skills has fostered a greater awareness of the substantial hindrance posed by writing difficulties in various contexts, our understanding of these challenges has not advanced at a proportional pace.

## Previous research on writing difficulties

Research into writing difficulties experienced by individuals with various language or hearing disorders, such as dyslexia, DLD, aphasia, and congenital hearing impairment, sheds light on the intricate relationship between linguistic and cognitive challenges and the written mode of communication.

Investigations into writing challenges faced by individuals with dyslexia have primarily centered around deficient spelling skills, often arising from struggles with phoneme-to-grapheme correspondences. For a review, see Sumner et al. (2014). These struggles impede the accurate translation of auditory word aspects into written form. Alongside spelling difficulties, issues related to grammar, punctuation, organizational structure, and overall composition quality have been frequently documented—even after texts have been corrected for spelling and capitalization (Tops et al., 2013). These challenges are believed to stem from cognitive bottlenecks during the transcription phase of writing (McCutchen, 1996; Berninger et al., 2002). However, the conclusive evidence supporting this notion remains elusive. While some studies, such as Sterling et al. (1998) and Wengelin (2007), revealed associations between spelling-related dysfluencies and lexical attributes in texts composed by individuals with dyslexia, other studies, including those by Connelly and colleagues, did not identify any discernible variance in lexical diversity (Connelly et al., 2006) or in the frequency of spelling revisions (Sumner and Connelly, 2020) between dyslexic and non-dyslexic individuals. However, Sumner and Connelly did indeed note that spelling revisions constituted a higher proportion of the total number of revisions for the dyslexic writers. These inconsistencies between studies may partly result from variations in demographics, input modalities, tasks, and languages across studies. Another possible explanation for the relatively impaired structure and content observed in texts by many writers with dyslexia is, as suggested by Torrance et al. (2016), that their reading deficit, along with their limited reading experience, has influenced their prior learning.

Similar gaps in research exist for DLD, a condition characterized by significant oral language production, and in severe cases, comprehension difficulties. Tucci and Choi (2023) conducted a comprehensive review of DLD's effects on writing skills, revealing challenges encompassing handwriting, spelling, organization, cohesion, planning, and narrative quality. Weaker grammatical abilities and the production of writing samples comparable in length to peers with typical language development were also noted. However, the impacts of DLD on both higher-level processes such as planning, revision, organization, and writing speed, and word-level processes, such as morphology, and spelling remain underexplored, and the results are therefore once again inconclusive. For example, while Shen and Troia (2018) found that, before receiving a writing strategy intervention, their participants with DLD did not plan or revise their writing at all, Koutsoftas (2016) found no differences between children with and without DLD.

Studies on writing by individuals with acquired writing difficulties, arising from brain damage caused by stroke, trauma, or neurodegenerative diseases like dementia are limited, and have like DLD primarily been investigated through the lens of spoken language when it comes to studying language production. While recent research on writing by individuals with aphasia have offered some new and cautionsly promising insights into the possibilities presented by intervention and compensatory tools (Behrns et al., 2009; Johansson-Malmeling et al., 2020), dementia research has primarily centered on differentiating between speech and writing in relation to aging. Its main focus has been on disease identification and progression rather than intervention. Notably, for Alzheimer's disease, there remains a lack of consensus regarding whether writing deficits manifest in the early stages of the disease or whether writing abilities are affected only in the later stages of the disease. In this context, investigations into the writing process can serve as an inconspicuous means for early detection of atypical aging and offer deeper insights into the linguistic regression associated with it (Van Waes et al., 2017; Afonso et al., 2019).

Also in the case of congenitally deaf or hard of hearing (DHH) individuals, research has predominantly emphasized reading and spoken skills. Their disorder is not intrinsically linguistic in its nature, but they often struggle with literacy, and writing may present unique challenges. DHH individuals often struggle to transfer morphosyntactic structures from speech to writing (Arfé, 2015; Mayer et al., 2016; Breland et al., 2022) both due to the high cognitive cost of perceiving and translating auditory sounds to writing, and for some, due to the written language being a second language with different structures than their first, signed, language (Wolbers et al., 2014). As DHH children increasingly participate in mainstream classrooms, there is a growing demand for greater understanding and awareness of their unique writing challenges.

In summary, research into writing difficulties across various language disorders highlights the multifaceted nature of linguistic and cognitive challenges when transitioning to the written mode. While spelling and transcription struggles are evident among individuals with dyslexia, DLD, and acquired language disorders, congenital hearing impairment also pose distinct obstacles to successful writing. A deeper understanding of these challenges can guide effective interventions and support individuals in overcoming barriers to written communication.

## Discussion

As shown in previous sections, a significant void persists in the domain of language disorders concerning the intricacies of writing difficulties and the underlying processes. While extensive research has shed light on reading and spoken language impairments, investigations into writing remain notably limited, hindering our ability to recognize, comprehend, and address writing-related challenges across various disorders. It is imperative that we address this gap through comprehensive studies that delve, not only into the final products of writing—the texts—, but into the writing process itself, as this knowledge is pivotal for both recognizing difficulties and devising effective remediation strategies.

For example, the findings from research on individuals with dyslexia underline the importance of unraveling the complexities of writing, particularly the connection between transcription skills, spelling, and overall composition quality. Such insights can provide a solid foundation for the identification of key intervention points to alleviate writing challenges. Similarly, developmental language disorder (DLD) research has revealed distinct hurdles in writing, from morphosyntactic transfers to organization and planning. This field remains underexplored in terms of understanding the precise processes that contribute to these difficulties and developing tailored interventions.

Considering persons with acquired disorders, such as aphasia and dementia, who in most cases have previously been skilled writers, it is somewhat surprising that the landscape of writing challenges has been left largely uncharted for so long. An awakening interest in understanding the production processes through methods like keystroke logging, exemplifies the need for a deeper exploration of writing difficulties in these contexts. Interestingly, dementia research stands out in that the focus has predominantly been on identifying disease-related linguistic changes rather than designing interventions. This disparity highlights the large range of opportunities offered by studies of the writing processes, for our understanding of the various aspects of language difficulties.

Furthermore, as mentioned above, the congenitally DHH population faces unique obstacles when it comes to writing. While reading and spoken skills have garnered more attention, research suggests that morphosyntactic transfers from speech to writing present significant challenges. This underscores the importance of understanding the cognitive demands of transcription for this group and underscores the significance of addressing writing-related difficulties in diverse populations.

In conclusion, the scarcity of research on writing and writing processes in both developmental and acquired language disorders is a critical issue. Enhanced studies focusing on these areas are pivotal for recognizing, understanding, and addressing writing challenges effectively. A balanced approach that mirrors the attention given to reading and spoken language can uncover the intricacies of writing impairments, thus paving the way for targeted interventions that empower individuals to overcome barriers in written communication, while also revealing some of the nuanced mechanisms underlying unaffected written expression. The six articles in this Frontiers Research Topic offer a significant contribution to the intricate realm of writing difficulties across various disorders. Through meticulous investigation, employing methodologies such as keystroke logging and handwriting capturing, they dissect the mechanics and cognitive aspects of writing as well as methods for exploring them. As our understanding of the writing process thus deepens, we will be able to provide more comprehensive support to those navigating the complex landscape of language disorders.

## Author contributions

ÅW: Writing—original draft, Writing—review & editing. IH: Writing—review & editing. LV: Writing—review & editing.
